# Declining risk of heart failure hospitalization following first acute myocardial infarction in Scotland between 1991–2016

**DOI:** 10.1002/ejhf.2965

**Published:** 2023-07-17

**Authors:** Kieran F. Docherty, Alice M. Jackson, Mark Macartney, Ross T. Campbell, Mark C. Petrie, Marc A. Pfeffer, John J.V. McMurray, Pardeep S. Jhund

**Affiliations:** ^1^ BHF Cardiovascular Research Centre University of Glasgow Glasgow UK; ^2^ Public Health Scotland Edinburgh UK; ^3^ Cardiovascular Division, Brigham & Women's Hospital Harvard Medical School Boston MA USA

**Keywords:** Heart failure, Myocardial infarction, Epidemiology

## Abstract

**Aim:**

Mortality from acute myocardial infarction (AMI) has declined, increasing the pool of survivors at risk of later development of heart failure (HF). However, coronary reperfusion limits infarct size and secondary prevention therapies have improved. In light of these competing influences, we examined long‐term trends in the risk of HF hospitalization (HFH) following a first AMI occurring in Scotland over 25 years.

**Methods and results:**

All patients in Scotland discharged alive after a first AMI between 1991 and 2015 were followed until a first HFH or death until the end of 2016 (minimum follow‐up 1 year, maximum 26 years). A total of 175 672 people with no prior history of HF were discharged alive after a first AMI during the period of study. A total of 21 445 (12.2%) patients had a first HFH during a median follow‐up of 6.7 years. Incidence of HFH (per 1000 person‐years) at 1 year following discharge from a first AMI decreased from 59.3 (95% confidence interval [CI] 54.2–64.7) in 1991 to 31.3 (95% CI 27.3–35.8) in 2015, with consistent trends seen for HF occurring within 5 and 10 years. Accounting for the competing risk of death, the adjusted risk of HFH at 1 year after discharge decreased by 53% (95% CI 45–60%), with similar decreases at 5 and 10 years.

**Conclusion:**

The incidence of HFH following AMI in Scotland has decreased since 1991. These trends suggest that better treatment of AMI and secondary prevention are having an impact on the risk of HF at a population level.

## Graphical abstract

Trends in the incidence of heart failure hospitalization following first myocardial infarction in Scotland 1991–2016. HF, heart failure; MI, myocardial infarction.

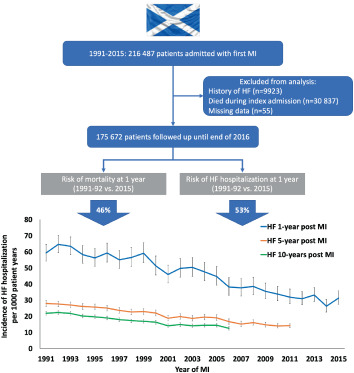

## Introduction

The last three decades have seen a substantial decline in the risk of mortality following acute myocardial infarction (AMI).^1^ It has been suggested that the increasing pool of AMI survivors, coupled with ageing of the population, may be contributing to a growing prevalence of heart failure (HF) in the general population.^2^, ^3^, ^4^ However, the widespread implementation of emergency coronary reperfusion services and improvements in secondary preventative therapy may have reduced individual risk of developing HF and offset any increase in the number of cases of incident HF related to greater survival after AMI.^5^ Further complicating this issue are the changing demographics of patients with AMI. The proportion of AMI presenting as ST‐elevation myocardial infarction (STEMI) has declined and the proportion of non‐ST elevation myocardial infarction (NSTEMI) has increased.^6^, ^7^, ^8^, ^9^ Although STEMI is typically associated with greater myocardial damage than NSTEMI, patients presenting with NSTEMI are frequently older and have a higher prevalence of comorbidities such as diabetes mellitus and chronic kidney disease which may contribute to an increased risk of developing HF.^10^ A further consideration contributing to the uncertainty about current trends is the increased availability of high‐sensitivity cardiac troponin assays which likely have led to the diagnosis of more cases of AMI with lesser degrees of myocardial injury. Prior epidemiological analyses have been limited in their ability to examine the effect of these factors on temporal trends of HF following AMI due to relatively short follow‐up periods, the inclusion of selected populations and the inclusion of patients with a history of HF (i.e. not examining the true incidence of HF following AMI).

To examine the complex interplay between these competing influences and their effect on trends in the long‐term risk of HF following AMI over the last quarter of a century, we examined the rates of first hospitalizations for HF in Scotland in patients who were discharged alive from a hospitalization for a first AMI between 1991 and 2015.

## Methods

### Data sources

Routinely collected clinical data on all discharges (including in‐hospital deaths and those patients discharged alive) from National Health Service (NHS) hospitals in Scotland are collated by Public Health Scotland (PHS) and the electronic Data Research and Innovation Service (eDRIS) of the NHS in Scotland. This study was approved by the NHS Scotland Public Benefit and Privacy Panel for Health and Social Care. Care is free at the point of delivery for all residents in Scotland, therefore these data represent virtually all hospitalizations in the country. The 2017 mid‐year population estimate in Scotland was 5 424 800. For each admission, information on discharge diagnoses (a principal diagnosis and up to five secondary diagnoses), procedures performed, demographics, prior admission diagnoses, postcode of residence and length of stay is recorded. Diagnoses are coded using the International Classification of Diseases (ICD) system (ICD 9th revision until April 1996 and ICD 10th revision thereafter). Record linkage is obtained through probability matching (with an accuracy of ≈98%) facilitating the analysis of data at the level of the individual patient and episode of care.^11^ Data on mortality are derived from linkage to the Registrar General's Death Certificate Data with an accuracy of 98%. When compared to adjudicated events in the setting of a clinical trial, the accuracy of discharge diagnoses in Scotland has been reported as >95%.^12^ Individual informed consent is not required for the data linkage.

For this study, we identified individuals aged 20 years or above with a first discharge from hospital with a principal diagnosis of AMI (ICD 9th revision 410 or ICD 10th revision I21 or I22). A first discharge was defined as one with an AMI code in the primary diagnostic position, with no prior hospitalization for AMI (in any diagnostic position) since 1981 (a minimum ‘look back’ of 9 years), the time‐point at which routine discharge coding was first available in Scotland. Patients with a history of HF recorded before their index AMI admission were excluded from this analysis, as were patients who died during their index AMI admission. A subsequent first HF hospitalization was defined as one occurring after discharge from the index AMI with a HF code (ICD 9th revision 425, 428, 402, or ICD 10th revision I50, I42, I11.0) in the primary diagnostic position.

Patients were allocated by postcode of residence into deprivation categories using the Scottish Index of Multiple Deprivation (SIMD) 2016 release, which takes into account seven measures of deprivation: current income, employment, health, education, housing, access to services and crime.^13^ Comorbid diagnoses were defined as those which were coded as a secondary diagnosis during hospitalization or as the principal diagnosis during a prior hospitalization within 5 years of the index hospitalization. The following comorbidities of interest were included in this analysis: coronary heart disease, hypertension, HF recorded during index AMI admission, atrial fibrillation, cerebrovascular disease, diabetes, peripheral vascular disease, chronic kidney disease, cancer, and respiratory disease. Information on procedures (percutaneous coronary intervention and/or coronary artery bypass grafting) was collected for those performed within 30 days of the index AMI.

### Statistical analysis

Baseline demographics are presented grouped by the year of the first admission with an AMI and whether patients had a subsequent hospitalization for HF. We report normally distributed continuous variables as means with standard deviations and skewed continuous variables as medians with interquartile ranges (IQR). Categorical variables are presented as counts and percentages.

Time‐to‐first hospitalization for HF was calculated as the time from discharge from a first AMI to a first admission with HF, or time to death from any cause or censoring on 31 December 2016 if never hospitalized for HF. To ensure a minimum of 1 year follow‐up for all patients, survival analysis was performed only on those patients with a first AMI from 1 January 1991 to 31 December 2015. Time to death was calculated as the time from discharge from a first admission with an AMI to death from any cause or censoring on 31 December 2016.

A joinpoint regression model was fitted to explore points of significant change in the trend of the incidence of admissions with AMI and to provide the estimated annual percentage change (Joinpoint Software, Version 4.6).^14^ The Bayesian information criterion was used to select the best‐fitting model. A maximum of 5 joinpoints were allowed for estimations and 95% confidence intervals (CI) were calculated for each estimate.

Crude incidence rates per 1000‐patient years were calculated for a first hospitalization for HF at 1, 5 and 10 years following discharge from index AMI and stratified by age, sex, deprivation (SIMD 2016 quintile), comorbidity, procedures performed and year of admission with AMI. To take into account temporal trends in the competing risk of death, the cumulative incidence rates of first HF hospitalization, stratified by year of index AMI, were calculated and presented using cumulative incidence curves, with the use of the non‐parametric cumulative incidence function of Fine and Gray with death from any cause treated as a competing risk.^15^ Competing risk regression models were used to explore the association of age, sex, deprivation (SIMD 2016 quintile), comorbidity, procedures performed and year of admission with AMI with HF hospitalization at 1, 5 and 10 years.

To examine the relative hazard for death following a first HF hospitalization, a Cox proportional hazards model was created in which a variable indicating HF hospitalization was entered into the model as a time‐updated covariate (with follow‐up time starting at discharge from index AMI) and adjusted for age, sex, deprivation (SIMD 2016 quintile), comorbidity, procedures performed and year of admission with AMI. The period at risk before a first HF hospitalization was attributed to the group with no HF hospitalization to calculate incidence rates which reflect patients' time‐updated event status. This was presented graphically using Kaplan–Meier estimates. The rate of death was calculated per 1000 patient‐years of follow‐up, with follow‐up starting on the day of the first HF hospitalization (or discharge from index AMI if the individual did not have a HF hospitalization). Temporal trends in mortality at 1 year following a first HF hospitalization were examined in a Cox‐proportional hazards model adjusting for the same variables as above with the exception of the year of AMI which was replaced by the year of first HF hospitalization and age at the time of HF hospitalization was included. Time to event was calculated as the time from admission with a first HF hospitalization to death or censoring at 1 year. Only those HF hospitalizations occurring up to the 31 December 2015 were included to ensure 1‐year follow‐up for all patients.

For patients who presented with a first AMI from 2012 to 2016, additional analyses using the methods described above were performed according to the classification of myocardial infarction presentation denoted by discharge coding (STEMI, NSTEMI, or unspecified).^16^

All analyses were performed using Stata 16 (StataCorp LP, College Station, TX, USA).

## Results

There were 216 487 patients admitted to hospital in Scotland with a first diagnosis of AMI between 1991 and 2015. After excluding those who had a history of HF before index admission (*n* = 9923), those who died during index admission (*n* = 30 837), and those with missing follow‐up data (*n* = 55), 175 672 patients were included in the cohort for analysis, providing 1.5 million patient‐years of follow‐up (online supplementary *Figure* *S1*).

### Trends in age‐standardized incidence of first acute myocardial infarction

**Figure 1:**
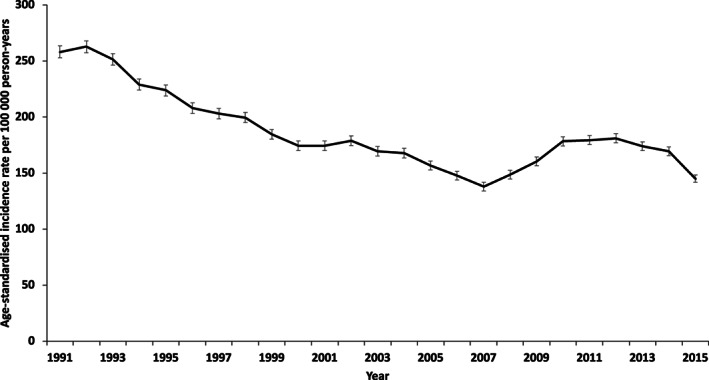
Trends in age‐standardized incidence of acute myocardial infarction 1991–2015.

*Figure* *1* shows the age‐standardized incidence of first AMI from 1991 to 2015. Overall, the annual rate decreased by 2.3% per year (95% CI 1.3–3.2; *p* < 0.001). We found significant differences in the trends in incidence of AMI over the time period examined; between 1991 and 2007 the rate of AMI decreased by 3.7% per year (95% CI 3.3–4.1; *p* < 0.001). The rate of AMI then increased between 2007 and 2012 by 5.9% per year (95% CI 2.1–9.7; *p* = 0.004). Subsequently, between 2012 and 2015, the trend was again for a decline in the rate of AMI with an annual decrease of 7.6% (95% CI 2.5–12.4; *p* = 0.007). The adjusted risk of death 1 year after AMI fell by 46% (95% CI 40–52; *p* < 0.001) between 1991 and 2015. The risk of death at 5 and 10 years after AMI fell by 37% (95% CI 33–41; *p* < 0.001) and 36% (95% CI 33–39; *p* < 0.001), respectively.

### Heart failure hospitalization following acute myocardial infarction

Of those patients who were discharged alive from a first AMI with no history of HF before their index admission, 21 445 (12.2%) were subsequently hospitalized for HF over a median follow‐up time of 6.7 years (IQR 2.8–12.9) (*Table* *1* and online supplementary *Table* *S1*). The median time from AMI to the development of HF was 2.6 years (IQR 0.4–7.7). In those who were subsequently admitted with HF, age at the time of AMI rose from 67.4 ± 10.9 in 1991–1995 to 74.7 ± 12.0 in 2011–2015; a similar pattern was not observed in those who did not develop HF (64.9 ± 12.2 [1991–1995] vs. 65.6 ± 13.6 [2011–2015]) (*Table* *1*). The mean age of patients at the time of first HF hospitalization within 1 year of discharge rose from 70.8 ± 10.7 to 76.2 ± 11.9 years (1991 vs. 2015); in men, age rose from 68.1 ± 10.6 to 73.6 ± 12.6 years and in women from 74.0 ± 9.8 to 79.5 ± 10.0 years. Similar increases were observed overall for HF occurring within 5 and 10 years (71.6 ± 10.7 to 76.8 ± 11.9 [1991 vs. 2011] and 72.3 ± 10.4 to 76.0 ± 11.8 [1991 vs. 2006], respectively).

**Table 1:** Baseline characteristics according to year of index acute myocardial infarction and occurrence of subsequent heart failure hospitalization

	1991–1995 (*n* = 43 592)		1996–2000 (*n* = 34 773)		2001–2005 (*n* = 31 032)		2006–2010 (*n* = 29 997)		2011–2015 (*n* = 36 278)	
	No HF (*n* = 35 189)	HF (*n* = 8403)	No HF (*n* = 29 359)	HF (*n* = 5414)	No HF (*n* = 27 463)	HF (*n* = 3569)	No HF (*n* = 27 646)	HF (*n* = 2351)	No HF (*n* = 34 570)	HF (*n* = 1708)
Age, years	64.9 ± 12.2	67.4 ± 10.9	65.0 ± 12.7	69.6 ± 11.2	65.5 ± 13.4	71.6 ± 11.7	65.5 ± 13.6	73.4 ± 11.8	65.6 ± 13.6	74.7 ± 12.0
Age group, years, *n* (%)										
<55	7265 (20.7)	1048 (12.5)	6344 (21.6)	577 (10.7)	6107 (22.2)	333 (9.3)	6543 (23.7)	174 (7.4)	8062 (23.3)	115 (6.7)
55–65	9343 (26.6)	2130 (25.4)	7360 (25.1)	1051 (19.4)	6489 (23.6)	550 (15.4)	6510 (23.6)	313 (13.3)	8295 (24.0)	204 (11.9)
65–74	10 543 (30.0)	2908 (34.6)	8500 (29.0)	1822 (33.7)	7187 (26.2)	1068 (29.9)	6583 (23.8)	631 (26.8)	8270 (23.9)	420 (24.6)
75–84	6428 (18.3)	1946 (23.2)	5430 (18.5)	1545 (28.5)	5651 (20.6)	1214 (34.0)	5700 (20.6)	861 (36.6)	6947 (20.1)	591 (34.6)
≥85	1610 (4.6)	371 (4.4)	1725 (5.9)	419 (7.7)	2029 (7.4)	404 (11.3)	2310 (8.4)	372 (15.8)	2996 (8.7)	378 (22.1)
Men, *n* (%)	22 015 (62.6)	4902 (58.3)	18 519 (63.1)	3092 (57.1)	17 391 (63.3)	1934 (54.2)	17 840 (64.5)	1251 (53.2)	22 210 (64.3)	937 (54.9)
Length of stay, days, median (IQR)	7 (6–10)	8 (7–12)	7 (5–9)	8 (6–12)	6 (5–9)	8 (6–13)	5 (4–9)	8 (5–14)	4 (3–7)	7 (4–13)
Deprivation category^a^, *n* (%)										
1 (most deprived)	9926 (28.5)	2508 (30.1)	7947 (27.2)	1550 (28.7)	7236 (26.5)	987 (27.7)	6657 (24.3)	597 (25.5)	8360 (24.5)	441 (25.9)
2	8813 (25.3)	2121 (25.5)	7177 (24.6)	1382 (25.6)	6609 (24.2)	875 (24.5)	6209 (22.7)	579 (24.7)	7843 (23.0)	444 (26.1)
3	6836 (19.6)	1698 (20.4)	5741 (19.7)	1100 (20.4)	5351 (19.6)	763 (21.4)	5589 (20.4)	457 (19.5)	7043 (20.6)	362 (21.3)
4	5091 (14.6)	1100 (13.2)	4594 (15.7)	785 (14.6)	4416 (16.2)	523 (14.7)	4956 (18.1)	404 (17.2)	5905 (17.3)	264 (15.5)
5 (least deprived)	4181 (12.0)	906 (10.9)	3727 (12.8)	579 (10.7)	3690 (13.5)	418 (11.7)	4001 (14.6)	308 (13.1)	5028 (14.7)	192 (11.3)
Comorbidity, *n* (%)										
Known coronary heart disease	4870 (13.8)	1367 (16.3)	5762 (19.6)	1361 (25.1)	7435 (27.1)	1148 (32.2)	12 153 (44.0)	1138 (484)	18 251 (52.8)	921 (53.9)
Hypertension	2645 (7.5)	800 (9.5)	4060 (13.8)	967 (17.9)	7162 (26.1)	1186 (33.2)	8997 (32.5)	1041 (44.3)	11 277 (32.6)	789 (46.2)
HF during index AMI admission	3211 (9.1)	1512 (18.0)	3296 (11.2)	1291 (23.9)	4293 (15.6)	1130 (31.7)	3758 (13.6)	732 (31.1)	3804 (11.0)	553 (32.4)
Atrial fibrillation	1530 (4.4)	537 (6.4)	1807 (6.2)	577 (10.7)	2238 (8.2)	549 (15.4)	2426 (8.8)	474 (20.2)	3328 (9.6)	429 (25.1)
Cerebrovascular disease	1367 (3.9)	371 (4.4)	1377 (4.7)	314 (5.8)	1393 (5.1)	262 (7.3)	1286 (4.7)	189 (8.0)	1509 (4.4)	132 (7.7)
Diabetes	2092 (6.0)	874 (10.4)	2446 (8.3)	832 (15.4)	2987 (10.9)	752 (21.1)	3376 (12.2)	618 (26.3)	5102 (14.8)	517 (30.3)
Peripheral arterial disease	1520 (4.3)	449 (5.3)	1455 (5.0)	369 (6.8)	1584 (5.8)	299 (8.4)	1639 (5.9)	274 (11.7)	2161 (6.3)	200 (11.7)
Chronic kidney disease	380 (1.1)	117 (1.4)	604 (2.1)	178 (3.3)	998 (3.6)	252 (7.1)	1410 (5.1)	318 (13.5)	2634 (7.6)	361 (21.1)
Cancer	1350 (3.8)	307 (3.7)	1329 (4.5)	242 (4.5)	1532 (5.6)	207 (5.8)	1593 (5.8)	152 (6.5)	2128 (6.2)	139 (8.1)
Respiratory disease	1992 (5.7)	526 (6.3)	2260 (7.7)	476 (8.8)	2842 (10.4)	415 (11.6)	3225 (11.7)	348 (14.8)	4870 (14.1)	335 (19.6)
Index MI procedures, *n* (%)										
PCI at admission	1190 (3.4)	278 (3.3)	2049 (7.0)	300 (5.5)	4382 (16.0)	366 (10.3)	11 672 (42.2)	552 (23.5)	18 183 (52.6)	482 (28.2)
PCI within 30 days	1241 (3.5)	284 (3.4)	2237 (7.6)	319 (5.9)	4803 (17.5)	399 (11.2)	12 132 (43.9)	580 (24.7)	19 243 (55.7)	497 (29.1)
CABG at admission	121 (0.3)	35 (0.4)	192 (0.7)	40 (0.7)	387 (1.4)	53 (1.5)	642 (2.3)	69 (2.9)	861 (2.5)	41 (2.4)
CABG within 30 days	155 (0.4)	44 (0.5)	260 (0.9)	51 (0.9)	514 (1.9)	66 (1.8)	800 (2.9)	83 (3.5)	995 (2.9)	52 (3.0)

AMI, acute myocardial infarction; CABG, coronary artery bypass grafting; HF, heart failure; IQR, interquartile range; MI, myocardial infarction; PCI, percutaneous coronary intervention.

^a^ Data missing in 1403 (0.8%) of patients.

Compared to patients who were never hospitalized for HF, those who had a first HF hospitalization were older at the time of their index AMI, more frequently women, and had evidence of greater socioeconomic deprivation (*Table* *1*). A history of HF during index AMI admission, atrial fibrillation, diabetes, peripheral arterial disease, and chronic kidney disease was more prevalent in those who were subsequently hospitalized for HF and patients were less likely to have undergone a revascularization procedure within 30 days of their index AMI. Percutaneous coronary revascularization within 30 days was performed in 82.0% of STEMI, 40.9% of NSTEMI and 40.3% of unspecified AMI (data available from 2012 to 2015 only). The prevalence of comorbidities also increased over time both in those who were subsequently hospitalized for HF and those who were not.

### Trends in incidence of a first hospitalization for heart failure

**Figure 2:**
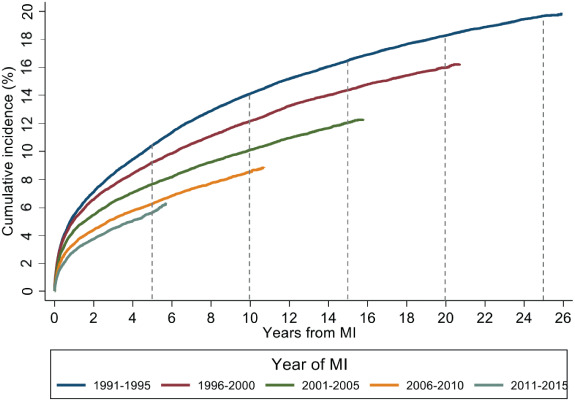
Cumulative incidence of first heart failure hospitalization following first acute myocardial infarction (MI) accounting for the competing risk of death from any cause. Data are presented by year of index acute MI. Dashed lines placed at 5‐year intervals following acute MI.

Crude case incidence (per 1000 patient‐years) for HF at 1 year following discharge from a first AMI fell from 59.3 (95% CI 54.2–64.7) in 1991 to 31.3 (27.3–35.8) in 2015 (*Figure* *2*). Similar trends were observed for HF occurring within 5 (27.9 [26.2–29.7] to 14.1 [12.8–15.5] – 1991 vs. 2011) and 10 years (21.8 [20.6–23.0] to 12.5 [11.4–13.6] – 1991 vs. 2006). Rates of HF hospitalization after 5 and 10 years in patients aged ≥85 years were 10‐fold higher than those aged <55 years (*Table* *2*). Rates were higher in women compared to men (10‐year rate: 22.1 [21.6–22.6] vs. 14.0 [13.7–14.2]). The rate of HF hospitalization increased with increasing socioeconomic deprivation – the rate of 10‐year HF hospitalization in the most deprived was 18.2 (17.7–18.7) compared to 14.1 (13.5–14.7) in the least deprived.

**Table 2:** Crude case incidence rates of heart failure following discharge after a first acute myocardial infarction

	HF incidence per 1000 patient‐years (95% confidence intervals)		
	1 year	5 year	10 year
All patients	47.0 (45.9–48.0)	20.7 (20.3–21.0)	16.8 (16.5–17.0)
Age, years			
<55	13.7 (12.6–15.0)	6.0 (5.6–6.4)	5.0 (4.8–5.3)
55–65	27.4 (25.9–29.1)	11.7 (11.2–12.2)	9.8 (9.5–10.2)
65–74	51.1 (49.1–53.3)	22.9 (22.2–23.6)	19.5 (19.0–20.0)
75–84	84.9 (81.7–88.2)	42.0 (40.8–43.2)	36.9 (36.0–37.9)
≥85	109.8 (103.5–116.5)	61.0 (58.2–63.9)	55.2 (52.8–57.8)
Sex			
Men	38.5 (37.3–39.7)	17.1 (16.7–17.5)	14.0 (13.7–14.2)
Women	61.7 (59.8–63.8)	27.3 (26.6–27.9)	22.1 (21.6–22.6)
Deprivation category			
1 (most deprived)	51.0 (48.9–53.2)	22.7 (22.0–23.4)	18.2 (17.7–18.7)
2	48.3 (46.1–50.5)	21.7 (21.0–22.5)	17.8 (17.3–18.3)
3	47.4 (45.1–49.8)	21.0 (20.2–21.8)	17.1 (16.6–17.7)
4	42.7 (40.3–45.3)	18.7 (17.9–19.5)	15.2 (14.6–15.8)
5 (least deprived)	42.7 (40.0–45.6)	17.6 (16.8–18.5)	14.1 (13.5–14.7)
Comorbidity			
Coronary heart disease	46.9 (45.1–48.9)	22.0 (21.4–22.7)	18.8 (18.3–19.3)
Hypertension	55.2 (52.8–57.7)	25.5 (24.7–26.4)	21.3 (20.6–21.9)
HF during index AMI admission	132.3 (127.3–137.5)	56.3 (54.6–58.0)	44.5 (43.3–45.8)
Atrial fibrillation	100.3 (94.7–106.3)	50.7 (48.5–52.9)	43.8 (42.1–45.6)
Cerebrovascular disease	81.9 (75.4–89.0)	41.6 (39.1–44.3)	35.0 (33.0–37.0)
Diabetes	83.6 (79.4–88.0)	41.4 (39.9–43.0)	35.3 (34.1–36.5)
Peripheral arterial disease	82.9 (77.0–89.3)	40.6 (38.3–42.9)	34.0 (32.3–35.8)
Chronic kidney disease	119.3 (110.7–128.5)	62.6 (58.9–66.4)	54.2 (51.2–57.4)
Cancer	64.5 (58.9–70.6)	31.1 (29.0–33.4)	26.4 (24.8–28.2)
Respiratory disease	60.2 (56.5–64.3)	28.8 (27.4–30.2)	24.3 (23.3–25.4)
Index MI procedures			
PCI or CABG within 30 days	20.5 (19.2–21.9)	9.0 (8.6–9.5)	8.2 (7.9–8.6)
No PCI or CABG within 30 days	56.7 (55.3–58.0)	24.9 (34.3–25.3)	19.4 (19.1–19.7)

AMI, acute myocardial infarction; CABG, coronary artery bypass grafting; HF, heart failure; MI, Myocardial infarction; PCI, percutaneous coronary intervention.

Accounting for the competing risk of death, the cumulative incidence of first HF hospitalization at 1 year fell between 1991 and 2015 from 5.3% to 2.9%; the 5‐year risk fell from 10.4% to 5.8% (1991 vs. 2011); the 10‐year risk from 14.4% to 9.0% (1991 vs. 2006) (*Figure* *2*). After adjustment for age, sex, socioeconomic deprivation, comorbidities and revascularization procedures, and accounting for the competing risk of death, the risk of HF hospitalization at 1 year after discharge fell by 53% (95% CI 45–60%) (*Table* *3*). The adjusted 5‐year risk of HF hospitalization fell by 57% (95% CI 52–61%) and the 10‐year risk fell by 48% (95% CI 44–53%). The 10‐year risk of HF following AMI was higher in older individuals (<55 vs. ≥85 years; hazard ratio [HR] 3.14; 95% CI 2.89–3.41), if there was HF complicating the index admission (HR 2.08; 95% CI 2.00–2.16), in patients with diabetes (HR 1.72; 95% CI 1.64–1.81), chronic kidney disease (HR 1.24; 95% CI 1.14–1.35), atrial fibrillation (HR 1.29; 95% CI 1.22–1.36), and in those not undergoing coronary revascularization within 30 days of AMI (HR 1.18; 95% CI 1.10–1.27). Similar results were seen at 1 and 5 years (*Table* *3*). The annual percent change in the risk of HF at 1 year following AMI between 1991 and 2015 was generally similar across subgroups of baseline characteristics (online supplementary Table *S2*) with the exception of a lesser reduction in risk with increasing age (interaction *p* = 0.001), in patients with atrial fibrillation (interaction *p* = 0.002) and in patients with chronic kidney disease (interaction *p* = 0.01).

**Table 3:** Adjusted risk of first heart failure hospitalization

	1 year	5 year	10 year
Year of AMI			
1991–1992	1.00	1.00	1.00
1993–1994	0.95 (0.87–1.04)	0.93 (0.87–0.99)	0.94 (0.89–0.99)
1995–1996	0.86 (0.78–0.94)	0.86 (0.81–0.92)	0.85 (0.80–0.90)
1997–1998	0.81 (0.74–0.90)	0.77 (0.72–0.83)	0.77 (0.72–0.82)
1999–2000	0.76 (0.69–0.84)	0.71 (0.66–0.76)	0.70 (0.65–0.74)
2001–2002	0.61 (0.55–0.68)	0.57 (0.53–0.62)	0.57 (0.53–0.61)
2003–2004	0.61 (0.55–0.68)	0.55 (0.50–0.59)	0.55 (0.51–0.59)
2005–2006	0.52 (0.46–0.58)	0.49 (0.45–0.53)	0.52 (0.47–0.56)^a^
2007‐2008	0.52 (0.46–0.58)	0.45 (0.41–0.49)	–
2009–2010	0.47 (0.42–0.53)	0.43 (0.39–0.48)	–
2011–2012	0.43 (0.38–0.48)	–	–
2013–2014	0.44 (0.38–0.49)	–	–
2015	0.47 (0.40–0.55)	–	–
Women vs. men	1.13 (1.08–1.18)	1.09 (1.05–1.13)	1.07 (1.04–1.11)
Age, years			
<55	1.00	1.00	1.00
55–64	1.71 (1.54–1.90)	1.65 (1.52–1.78)	1.60 (1.50–1.72)
65–74	2.63 (2.39–2.91)	2.58 (2.39–2.78)	2.48 (2.33–2.65)
75–84	3.76 (3.40–4.15)	3.76 (3.49–4.06)	3.36 (3.14–3.60)
≥85	4.32 (3.86–4.84)	3.98 (3.65–4.35)	3.14 (2.89–3.41)
Deprivation category			
1 (most deprived)	1.00	1.00	1.00
2	0.90 (0.84–0.96)	0.91 (0.86–0.95)	0.92 (0.88–0.96)
3	0.89 (0.83–0.95)	0.88 (0.84–0.93)	0.92 (0.88–0.97)
4	0.81 (0.76–0.87)	0.81 (0.77–0.86)	0.83 (0.78–0.87)
5 (least deprived)	0.80 (0.74–0.87)	0.77 (0.72–0.82)	0.77 (0.73–0.82)
Comorbidity			
Coronary heart disease	1.11 (1.06–1.17)	1.17 (1.12–1.22)	1.16 (1.11–1.21)
Hypertension	1.09 (1.03–1.16)	1.12 (1.07–1.17)	1.11 (1.06–1.16)
HF during index AMI admission	2.70 (2.57–2.84)	2.31 (2.22–2.41)	2.08 (2.00–2.16)
Atrial fibrillation	1.36 (1.28–1.45)	1.34 (1.27–1.42)	1.29 (1.22–1.36)
Cerebrovascular disease	1.04 (0.95–1.14)	1.04 (0.97–1.12)	1.01 (0.94–1.09)
Diabetes	1.67 (1.57–1.77)	1.76 (1.68–1.85)	1.72 (1.64–1.81)
Peripheral arterial disease	1.26 (1.17–1.37)	1.23 (1.15–1.31)	1.16 (1.09–1.24)
Chronic kidney disease	1.58 (1.45–1.71)	1.44 (1.34–1.55)	1.24 (1.14–1.35)
Cancer	0.98 (0.89–1.07)	0.87 (0.81–0.95)	0.85 (0.78–0.92)
Respiratory disease	1.14 (1.06–1.22)	1.11 (1.05–1.18)	1.07 (1.01–1.13)
Index MI procedures			
No PCI or CABG within 30 days	1.45 (1.34–1.57)	1.41 (1.32–1.50)	1.18 (1.10–1.27)

Data presented as hazard ratio (95% confidence intervals) adjusted for age, comorbidities, socioeconomic deprivation, and year of admission.

AMI, acute myocardial infarction; CABG, coronary artery bypass grafting; HF, heart failure; MI, myocardial infarction; PCI, percutaneous coronary intervention.

^a^ Includes only patients with AMI in 2006.

### Myocardial infarction type

**Figure 3:**
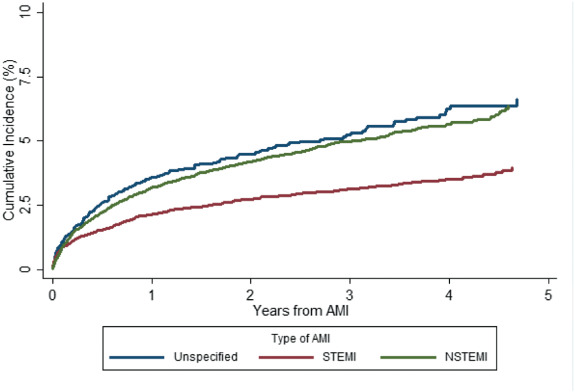
Cumulative incidence of first heart failure hospitalization following first acute myocardial infarction (AMI) by type of AMI (2012–2015) accounting for the competing risk of death from any cause. NSTEMI, non‐ST‐elevation myocardial infarction; STEMI, ST‐elevation myocardial infarction.

From 2012 to 2015 the rate of STEMI and NSTEMI could be calculated using specific codes introduced in Scotland for the coding of subtypes of myocardial infarction. Of the 29 011 myocardial infarctions occurring during this period, 10 148 (35.0%) were classified as STEMI, 16 456 (56.7%) as NSTEMI and 2407 (8.3%) were unspecified. During follow‐up from 1 January 2012 to 31 December 2016 (minimum 1 year and maximum 5 years), the incidence of hospitalization for HF per 1000 person‐years was 11.3 (95% CI 10.1–12.6) following STEMI, 19.3 (18.0–20.6) after NSTEMI and 20.4 (17.2–24.1) after unspecified myocardial infarction; the cumulative incidence of a first hospitalization for HF is displayed by type of myocardial infarction in *Figure* *3*. Accounting for the competing risk of death from any cause and adjusting for age, deprivation, comorbidities, year of AMI and revascularization, compared to STEMI, the HR of HF hospitalization was 1.01 (95% CI 0.87–1.16) for NSTEMI and 1.04 (95% CI 0.83–1.29) for unspecified myocardial infarction.

### Mortality

**Figure 4:**
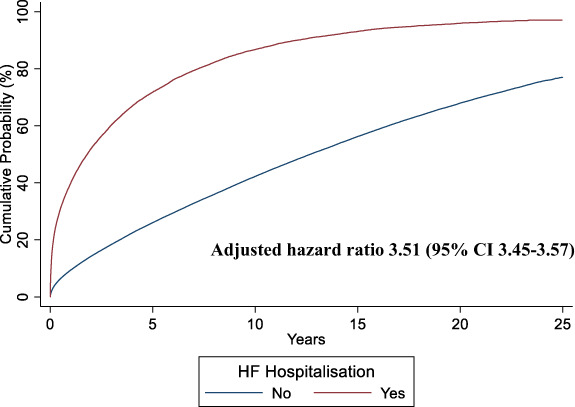
Kaplan–Meier curves for death from any cause following a first heart failure (HF) hospitalization or time from acute myocardial infarction discharge if no HF hospitalization occurred.

Annualized mortality was five‐fold greater in those after a first hospitalization for HF compared to those who were never hospitalized for HF; the fatality rate per 1000 patient‐years was 254.2 (95% CI 250.5–258.0) for patients following a first HF hospitalization and 53.7 (53.3–54.1) for those never hospitalized for HF following a first AMI. When considered as a time‐updated covariate, the occurrence of a first hospitalization for HF was associated with an over three‐fold risk of death compared to those who never had this event (adjusted HR 3.51; 95% CI 3.45–3.57) (*Figure* *4*). Following a first hospitalization for HF, median survival did not change significantly between 1991–1995 and 2011–2016 (1.7 years [95% CI 1.5–1.8] vs. 1.8 years [1.7–1.9]). However, after covariate adjustment, the risk of death at 1 year following a first hospitalization for HF fell by 30% (95% CI 15–43%) between 1991 and 2015. This represented a yearly decrease in the risk of mortality following admission with HF by 1.3% (95% CI 1.0–1.6%; *p* < 0.001).

## Discussion

In this nationwide study of a population of 5.4 million individuals with a single healthcare provider, we report that the incidence of a first hospitalization for HF following discharge after a first AMI decreased over the last quarter of a century (*Graphical Abstract*). This was despite a progressive reduction in the risk of mortality following AMI, increasing the potential pool of survivors at risk of developing HF, with increasing age and prevalence of comorbidities such as diabetes among these survivors.

Both the short‐ and long‐term risk of mortality following AMI have fallen over the last quarter century, likely as a result of an increase in the availability of coronary reperfusion services, improvements in secondary prevention, and increasing sensitivity of troponin assays and diagnosis of AMI with lesser degrees of myocardial injury (and lower subsequent risk of mortality).^1^ In the present study, along with these improvements in survival, we identified three trends which could potentially drive an increasing incidence of HF following a first AMI. Firstly, age at the time of AMI (and at the time of first HF hospitalization) increased and secondly, the prevalence of comorbidities associated with a higher risk of developing HF following AMI such as diabetes and chronic kidney disease rose in AMI patients.^10^, ^17^, ^18^ Another trend that might have led to an increase in the rate of incident HF was the increase in the proportion of NSTEMI to STEMI, as HF was more likely to develop after NSTEMI than STEMI.^6^

Despite these observations, we found that the crude incidence of HF following AMI decreased over the period studied. Previous studies have reported similar findings in Olmsted County, United States (1990–2010),^19^ Sweden (1993–2004 and 2004–2013),^20^, ^21^ Western Australia (1996–2007),^22^ Denmark (2000–2017),^23^ England (1998–2010),^24^ in Medicare beneficiaries in the United States (1998–2010 and 2000–2013),^25^, ^26^ New Jersey, United States (2000–2015),^27^ and in Norway (2001–2009).^28^ The strength of our dataset is that the follow‐up covers more than a quarter century, including the pre‐ and post‐primary percutaneous coronary intervention eras, with 1.5 million patient‐years of follow‐up. Therefore, we were able to examine the influence of changes in patient characteristics, diagnostic criteria (change to high‐sensitivity troponin), AMI phenotype, therapy and practice, and long‐term survival following AMI on the subsequent risk of HF at a country‐wide level and in a single healthcare provider system. Most importantly, unlike most prior studies, including recent reports from Italy, and the United States, we were able to exclude patients with a history of HF, thus describing true incident cases.^26^, ^29^

What may explain this decrease? Firstly, the use of emergent coronary reperfusion procedures and revascularization has increased exponentially, thereby reducing infarct size and the subsequent risk of HF. This is supported by reports of a reduction in the incidence of HF complicating the index AMI admission, particularly in patients with STEMI, a factor which is greatly influenced by the degree of acute ventricular damage sustained at the time of AMI.^30^ Additionally, we suggest that increased uptake of secondary preventative therapies has contributed to a decreased long‐term risk of HF in two ways. Firstly, improvements in coronary stent technology along with the use of more effective anti‐platelet agents and statins, promote infarct artery patency and reduce the risk of both acute and remote reinfarction, thereby limiting myocardial damage.^31^, ^32^, ^33^ Secondly, inhibitors of the renin–angiotensin–aldosterone system and beta‐blockers reduce the risk of adverse left ventricular remodelling and therefore, the development of HF.^34^, ^35^, ^36^, ^37^ Other studies have shown that the implementation of these secondary preventative measures has increased over time and this has likely contributed to reducing the risk of developing HF in both the acute period following myocardial infarction and in the longer term.^10^, ^18^, ^38^

Elderly patients, as well as being at a greater risk of developing HF, also have a higher competing risk of death from any cause. This may, in part, explain why we have observed that an increase in the proportion of elderly patients with AMI has not translated into an increase (or plateauing) in the overall crude incidence of a first hospitalization for HF. However, population studies have reported an increasing prevalence of HF in the general population, secondary to a growing population of elderly patients. The present analyses highlight that increasing age is an independent risk factor for the development of HF and ongoing efforts should be made in the implementation of preventative therapies in elderly patients following AMI. A further factor which should be considered in the decreasing incidence of HF is the changes in the definition of AMI and increased use of high‐sensitivity troponin assays, resulting in a greater proportion of AMI representing relatively small infarcts with a subsequently low risk of developing HF.^39^, ^40^ We found evidence of an increase in the age‐standardized incidence of AMI from 2007 following the release of the universal definition of myocardial infarction^41^; we believe this reflects the increased use of troponin and high‐sensitivity assays to diagnose AMI, along with the introduction of the definition of type 1 and type 2 myocardial infarction. Following this, the rate of myocardial infarction declined again, consistent with an overall downward trend in the rate of AMI observed in Scotland and globally. We found a higher cumulative incidence of HF following discharge for NSTEMI and unspecified myocardial infarction compared to STEMI, although, after adjustment for prognostic variables (including age, gender and comorbidities), there was no significant difference in the risk of HF between types of myocardial infarction. This finding likely reflects the different phenotypes of patients presenting with NSTEMI and STEMI, in that patients with NSTEMI are older with more frequent comorbidities and therefore more frequently develop HF following AMI. It may also reflect a degree of misdiagnosis with small troponin elevations related to myocardial injury in the setting of HF presentations being incorrectly classified as NSTEMI.

Over the period examined, median survival following a first HF hospitalization did not change significantly. However, after covariate adjustment, including the year of HF hospitalization and age at the time of hospitalization, the risk of death at 1 year had fallen by 30% (95% CI 15–43%) between 1991 and 2015. Advances in both pharmacological and device therapy for HF with reduced ejection fraction have contributed to the reduction in the risk of mortality.^42^, ^43^ The five‐fold greater annualized rate of mortality for patients following a first hospitalization for HF following AMI compared to those never hospitalized for HF should act as a reminder to physicians that prevention of this event should be one of the key priorities in care following AMI. Our analysis has highlighted that particular sub‐groups of patients are at a relatively increased risk of HF and require close attention to ensure the appropriate use of reperfusion service resources and implementation of secondary preventative measures. As already mentioned, elderly patients are at increased risk of developing HF but often do not receive reperfusion therapy and are less likely to be prescribed secondary preventative medications.^5^, ^9^, ^44^, ^45^ Similarly, patients with diabetes or chronic kidney disease are less likely to receive evidence‐based treatments, in particular revascularization, although, because patients with advanced chronic kidney disease are frequently excluded from clinical trials, evidence of benefit in this group is scarce.^46^, ^47^, ^48^ Recently, a new predictive tool has been described that may help identify the highest risk patients for targeted preventive therapy and new therapies are being tested.^5^, ^49^ Although the recent PARADISE‐MI trial (Prospective ARNI versus ACE Inhibitor Trial to Determine Superiority in Reducing Heart Failure Events after Myocardial Infarction) did not meet its primary endpoint, there was a nominally statistically significant reduction in total HF events and cardiovascular death suggesting there may be a role for sacubitril/valsartan in preventing HF after AMI, although this needs further investigation.^50^, ^51^ Ongoing trials with sodium–glucose cotransporter 2 (SGLT2) inhibitors in patients following AMI (ClinicalTrials.gov identifiers NCT04564742 and NCT04509674) will provide outcome data on the effect of these drugs in this high‐risk population in the context of recently reported favourable effects of SGLT2 inhibition on left ventricular remodelling when initiated early following AMI.^52^

Women were 12% more likely than men to develop HF at 1 year following AMI and this difference persisted out to 10 years (adjusted HR 1.07; 95% CI 1.04–1.11). Similar findings were reported in an analysis of the HORIZONS‐AMI trial (Harmonizing Outcomes with Revascularization and Stents in Acute Myocardial Infarction) where women were more likely to develop HF at 2 years following AMI (the multivariate adjusted odds ratio was 1.34, 95% CI 1.10–1.51) and in an analysis of high‐risk AMI patients (those in whom AMI was complicated by HF, left ventricular systolic dysfunction, or both) in the VALIANT trial (Valsartan in Acute Myocardial Infarction Trial).^53^, ^54^ Potential reasons which have suggested for this include that women are more likely to have a delayed presentation with non‐chest pain symptoms of AMI (potentially increasing infarct size) and are less likely to receive evidence‐based treatments (including revascularization) and effective secondary prevention than men.^55^, ^56^, ^57^, ^58^, ^59^ We were unable to directly examine these factors in our analysis. In the present study women, compared with men, were older at the time of AMI, had a greater prevalence of hypertension, atrial fibrillation, diabetes, renal impairment, more frequently had HF complicating index AMI admission and were less likely to have percutaneous revascularization within 30 days of AMI (18.3% vs. 27%). Despite adjustment for these factors, women remained at an independently increased risk relative to men which may reflect gender imbalances in AMI care along with other unmeasured confounders. Finally, in the setting of a universal, single healthcare provider, we have identified persisting differences in outcomes by level of socioeconomic deprivation. Although this finding is not novel, it does highlight the need to focus efforts to ensure equal provision of resources and robust follow‐up for those patients at greatest risk of developing HF.

As with all analyses of this nature, there are limitations. We did not have information on ejection fraction, cardiac biomarker levels or timing of reperfusion therapy or revascularization procedures which may influence the risk of development of HF. Furthermore, we did not have access to relevant laboratory or clinical data such as blood pressure or kidney function. We did not have information on secondary preventative therapies which may influence these trends. We only examined mortality from any cause as information regarding cause‐specific death from death certificates is unreliable. Our analysis of outcomes by type of myocardial infarction was limited to a restricted and recent time period following the implementation of specific sub‐classification coding in Scotland. We are unable to distinguish between type 1 and type 2 myocardial infarction. We do not have information regarding the community‐based diagnosis of HF, however the majority of HF diagnoses are made in the hospital setting.^60^, ^61^ We were unable to differentiate between presentations with HF with a reduced or preserved ejection fraction. Finally, our study examined the period before the COVID‐19 pandemic which led to decreases in hospital admissions with AMI in many countries and the consequences for future trends in HF and death are uncertain.^62^, ^63^

## Conclusion

Both the risk of mortality and the incidence of HF hospitalization following AMI in Scotland have consistently decreased since 1991. These trends suggest that better treatment of myocardial infarction and secondary prevention are having an impact on the risk of HF at a population level. Furthermore, changes in the diagnostic criteria of AMI, a decreasing incidence of STEMI and rising NSTEMI incidence may have resulted in a population at lower risk of HF because of less myocardial damage. The risk of mortality following a first hospitalization for HF also fell over the period examined, likely reflecting advances in the treatment of HF with reduced ejection fraction.

### Funding

This work was funded by an National Health Service Greater Glasgow and Clyde Endowment fund award (GN17CA406). Mark C. Petrie, John J.V. McMurray, and Pardeep S. Jhund are supported by British Heart Foundation Centre of Research Excellence (Grant RE/18/6/34217) and the Vera Melrose Heart Failure Research Fund.

**Conflict of interest**: K.F.D.'s employer, the University of Glasgow, has been remunerated by AstraZeneca for work on clinical trials; he has received speakers honoraria from AstraZeneca, Pharmacosmos and Radcliffe Cardiology, has served on an advisory board for Us2.ai and Bayer AG, served on a clinical endpoint committee for Bayer AG, and has received grant support from AstraZeneca, Boehringer Ingelheim, Novartis and Roche Diagnostics (paid to his institution). R.T.C. has served on an advisory board for Bayer AG. M.C.P. reports lecture fees from AstraZeneca and Eli Lilly; personal fees from Novo Nordisk, AstraZeneca, NAPP Pharmaceuticals, Takeda Pharmaceutical, Alnylam, Bayer, Resverlogix and Cardiorentis; and grants and personal fees from Boehringer Ingelheim and Novartis. M.A.P. reports research grant support to his institution from Novartis; is a consultant to AstraZeneca, Boehringer Ingelheim and Eli Lilly Alliance, Corvidia, DalCor, GlaxoSmithKline, Lexicon, NHLBI CONNECTs (Master Protocol Committee), Novartis, Novo Nordisk, Peerbridge and Sanofi; and has equity in DalCor. J.J.V.M. has received payments through Glasgow University for work on clinical trials; has received consulting fees and other activities from Alnylam, Amgen, AstraZeneca, Bayer, Boehringer Ingelheim, Bristol Myers Squibb, Cardurion, Cytokinetics, Dal‐Cor, GlaxoSmithKline, Ionis, KBP Biosciences, Novartis, Pfizer, and Theracos; has received personal lecture fees from the Corpus, Abbott, Hikma, Sun Pharmaceuticals, Medscape/Heart.Org, Radcliffe Cardiology, and Servier; and has served as Director of Global Clinical Trial Partners. P.S.J. reports speakers fees from AstraZeneca, Novartis, Alkem Metabolimics, ProAdWise Communications, Sun Pharmaceuticals, Intas Pharma; advisory board fees from AstraZeneca, Boehringer Ingelheim, Novartis; research funding from AstraZeneca, Boehringer Ingelheim, Analog Devices Inc, Roche Diagnostics. P.S.J's employer, the University of Glasgow, has been remunerated for clinical trial work from AstraZeneca, Bayer AG, Novartis and NovoNordisk. Director Global Clinical Trial Partners Ltd. All other authors have nothing to disclose.

## Supporting information


**Appendix S1.** Supporting Information.
