# Supplementary material for: Declining risk of heart failure hospitalization following first acute myocardial infarction in Scotland between 1991–2016
Source: Eur J Heart Fail. 2023 Jul 17;25(8):1213–24. doi: 10.1002/ejhf.2965 (PMC10946471; doi:10.1002/ejhf.2965)
Supplement: Supplementary file 1 — Appendix S1. Supporting Information. [file EJHF-25-1213-s001.docx]

**Declining risk of heart failure hospitalization following first acute myocardial infarction in Scotland between 1991-2016**

**Supplementary Material**

**Supplementary Table 1: Baseline characteristics according to development of HF**

|  | **No HF**  **(n=154277)** | **HF**  **(n=21445)** |
| --- | --- | --- |
| **Age - years** | 65.3 ± 13.1 | 69.9 ± 11.6 |
| **Age group – no. (%)** |  |  |
| <55 | 34321 (22.3) | 2247 (10.5) |
| 55-64 | 37997 (24.6) | 4248 (19.8) |
| 65-74 | 41083 (26.6) | 6849 (31.9) |
| 75-84 | 30156 (19.6) | 6157 (28.7) |
| ≥85 | 10670 (6.9) | 1944 (9.1) |
| **Men – no (%)** | 97975 (63.5) | 12116 (56.5) |
| **Median length of stay – days (IQR)** | 6 (4-9) | 8 (6-12) |
| **Deprivation category* – no. (%)** |  |  |
| 1 (most deprived) | 40126 (26.2) | 6083 (28.5) |
| 2 | 36651 (24.0) | 5401 (25.3) |
| 3 | 30560 (20.0) | 4380 (20.5) |
| 4 | 24962 (16.3) | 3076 (14.4) |
| 5 (least deprived) | 20627 (13.5) | 2403 (11.3) |
| **Co-morbidity – no. (%)** |  |  |
| Coronary heart disease | 48471 (31.4) | 5935 (27.7) |
| Hypertension | 34141 (22.1) | 4783 (22.3) |
| Heart failure during index AMI admission | 18362 (11.9) | 5218 (24.3) |
| Atrial fibrillation | 11329 (7.4) | 2566 (12.0) |
| Cerebrovascular disease | 6932 (4.5) | 1268 (5.9) |
| Diabetes | 16003 (10.4) | 3593 (16.8) |
| Peripheral arterial disease | 8359 (5.4) | 1591 (7.4) |
| Chronic kidney disease | 6026 (3.9) | 1226 (5.7) |
| Cancer | 7932 (5.1) | 1047 (4.9) |
| Respiratory disease | 15189 (9.9) | 2100 (9.8) |
| **Index MI procedures** |  |  |
| PCI during admission | 37476 (24.3) | 1978 (9.2) |
| PCI within 30 days | 39656 (25.7) | 2079 (9.7) |
| CABG during admission | 2203 (1.4) | 238 (1.1) |
| CABG within 30 days | 2724 (1.8) | 296 (1.4) |

*Data missing in 1403 (0.8%) of patients. Abbreviations: IQR, interquartile range; AMI, acute myocardial infarction; PCI, percutaneous coronary intervention; CABG, coronary artery bypass grafting

**Supplementary Table 2: Annual change in the risk of HF at 1 year following AMI between 1991 and 2015 by subgroups of baseline characteristics**

|  | **Annual percent change (95%) in risk of HF admission 1991-2015** | **Interaction P value** |
| --- | --- | --- |
| **Sex** |  | **0.34** |
| Men | - 3.9 (-4.5, -3.4) |  |
| Women | -4.1 (-4.6, -3.6) |  |
| **Age (years)** |  | **0.001** |
| <55 | -5.3 (-6.8 , - 3.9) |  |
| 55-64 | -4.4 (-5.4, -3.4) |  |
| 65-74 | -4.9 (-5.4, -4.2) |  |
| 75-84 | -3.9 (-4.5, -3.2) |  |
| >=85 | -1.9 (-2.9, -1.0) |  |
| **Atrial fibrillation** |  | **0.002** |
| Yes | -2.5 (-3.4, -1.6) |  |
| No | -4.3 (-4.7, -3.9) |  |
| **Cerebrovascular disease** |  | **0.64** |
| Yes | -3.7 (-5.0, -2.4) |  |
| No | -4.0 (-4.4, -3.6) |  |
| **Cancer** |  | **0.99** |
| Yes | -3.9 (-4.3, -1.4) |  |
| No | -4.1 (-4.5, -3.7) |  |
| **Diabetes** |  | **0.51** |
| Yes | -3.3 (-4.1, -2.5) |  |
| No | -4.2 (-4.6, -3.7) |  |
| **Hypertension** |  | **0.58** |
| Yes | -4.2 (-4.9, -3.4) |  |
| No | -4.0 (-4.4, -3.5) |  |
| **Peripheral arterial disease** |  | **0.55** |
| Yes | -3.3 (-4.4, -2.1) |  |
| No | -4.1 (-4.4, -3.7) |  |
| **Respiratory disease** |  | **0.10** |
| Yes | -5.4 (-6.4, -4.4) |  |
| No | -3.8 (-4.1, -3.3) |  |
| **Chronic kidney disease** |  | **0.01** |
| Yes | -2.1 (-34, -0.9) |  |
| No | -4.2 (-4.5, -3.8) |  |
| **Coronary heart disease** |  | **0.27** |
| Yes | -4.2 (-4.8, -3.6) |  |
| No | -3.9 (-4.3, -3.4) |  |

**Supplementary Figure 1: Study Population**

**
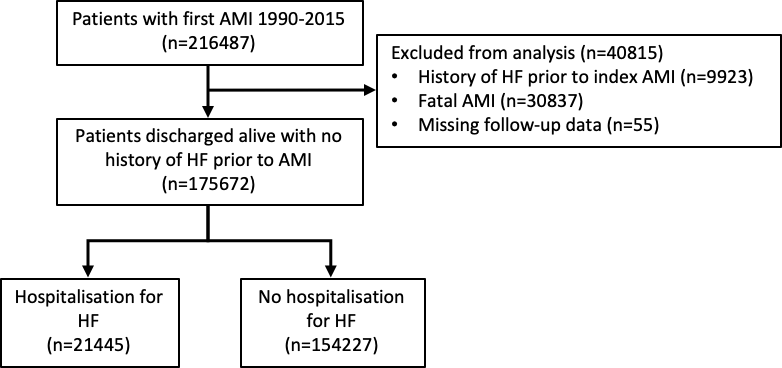
**

**Legend:** AMI, acute myocardial infarction; HF, heart failure.
